# Discovery of a highly selective KIT kinase primary V559D mutant inhibitor for gastrointestinal stromal tumors (GISTs)

**DOI:** 10.18632/oncotarget.22624

**Published:** 2017-11-15

**Authors:** Kailin Yu, Xuesong Liu, Zongru Jiang, Chen Hu, Fengming Zou, Cheng Chen, Juan Ge, Jiaxin Wu, Xiaochuan Liu, Aoli Wang, Wenliang Wang, Wenchao Wang, Ziping Qi, Beilei Wang, Li Wang, Hezhong Yan, Jiaoxue Wang, Tao Ren, Jun Tang, Qingsong Liu, Jing Liu

**Affiliations:** ^1^ High Magnetic Field Laboratory, Chinese Academy of Sciences, Hefei, Anhui 230031, P. R. China; ^2^ University of Science and Technology of China, Hefei, Anhui 230036, P. R. China; ^3^ Key Laboratory of High Magnetic Field and Ion Beam Physical Biology, Hefei Institutes of Physical Science, Chinese Academy of Sciences, Hefei, Anhui 230031, P. R. China; ^4^ Precision Targeted Therapy Discovery Center, Institute of Technology Innovation, Hefei Institutes of Physical Science, Chinese Academy of Sciences, Hefei, Anhui 230088, P. R. China; ^5^ Precision Medicine Research Laboratory of Anhui Province, Hefei, Anhui 230088, P. R. China; ^6^ Department of Gastroenterology, The 105th Hospital of People’s Liberation Army, Hefei, Anhui 230031, P. R. China

**Keywords:** KIT, KIT V559D, GISTs, primary mutations, secondary mutations

## Abstract

KIT kinase V559D mutation is the most prevalent primary gain-of-function mutation in Gastrointestinal Stromal Tumors (GISTs). Here we reported a highly selective KIT V559D inhibitor CHMFL-KIT-031, which displayed about 10-20 fold selectivity over KIT wt in the biochemical assay (IC_50_: 28 nM over 168 nM; Kd: 266 nM versus 6640 nM) and in cell (EC_50_: 176 nM versus 2000 nM for pY703) examination. It also displayed 15∼400-fold selectivity over other primary mutants such as L576P and secondary mutants including T670I, V654A (ATP binding pocket) as well as N822K and D816V (activation loop). In addition, it exhibited a selectivity S score (1) of 0.01 among 468 kinases/mutants in the KINOMEScan^™^ assay. CHMFL-KIT-031 showed potent inhibitory efficacy for KIT V559D mediated signaling pathways in cell and anti-tumor activity *in vivo* (Tumor Growth Inhibition: 68.5%). Its superior selectivity would make it a good pharmacological tool for further dissection of KIT V559D mediated pathology in the GISTs.

## INTRODUCTION

Gastrointestinal stromal tumors (GISTs) are the most common mesenchymal tumors of the gastrointestinal tract. About 80% GIST patients harbor KIT kinase primary gain of function mutations [1, 2]. The mutations that occur in the exon 11 of KIT gene which encodes the juxtamembrane domain (such as V559D, L576P) of KIT kinase account for the majority (about 70%) of the primary gain of function mutations and the mutations that occur in the exon 9 which encodes the extracellular dimerization domain account for 10-15%[3]. Secondary mutations usually occur in the kinase domain either in the ATP binding pocket (such as T670I and V654A) or the activation loops (such as N822K, D816V) after drug treatments. Clinical screening showed that KIT V559D mutant ranks first in the most frequent mutations in the exon 11 [4]. Gain-of-function mutation will mimic the growth factor (SCF) stimulated constitutive kinase activation thus leads to the activated downstream signaling pathways including PI3K/AKT, RAF/MEK/ERK which facilitated the tumorigenesis [5]. However, how single point mutations such as V559D leads to the kinase activation is still unclear.

Currently there are a number of KIT kinase inhibitors such as Imatinib [6], Sunitinib [7] and Regorafenib [8] which are approved for the first, second and third line clinical applications, as well as the preclinical research tool CHMFL-KIT-110 [9] etc. However, all of the currently clinically used drugs are multiple kinase target inhibitors, which limits their usage as chemical biology research tools. Even the most KIT kinase selective inhibitor CHMFL-KIT-110 could not achieve mutant selectivity among different KIT wt/mutants. In order to precisely determine the biological and pathological roles of gain-of-function mutants, highly mutant selective chemical research tool is highly desired. However, usually kinases with the activation mutations that occur in the non-ATP binding pocket are very hard for rational design of the inhibitors because the structural conformation change is not easily reflected in the X-ray 3D structures. Here we report the discovery of a highly KIT V559D primary mutant selective chemical biology research tool CHMFL-KIT-031 through a high throughput screening approach.

## RESULTS

### CHMFL-KIT-031 selectively inhibits the proliferation of BaF3-TEL-KIT-V559D cells

**Figure 1:**
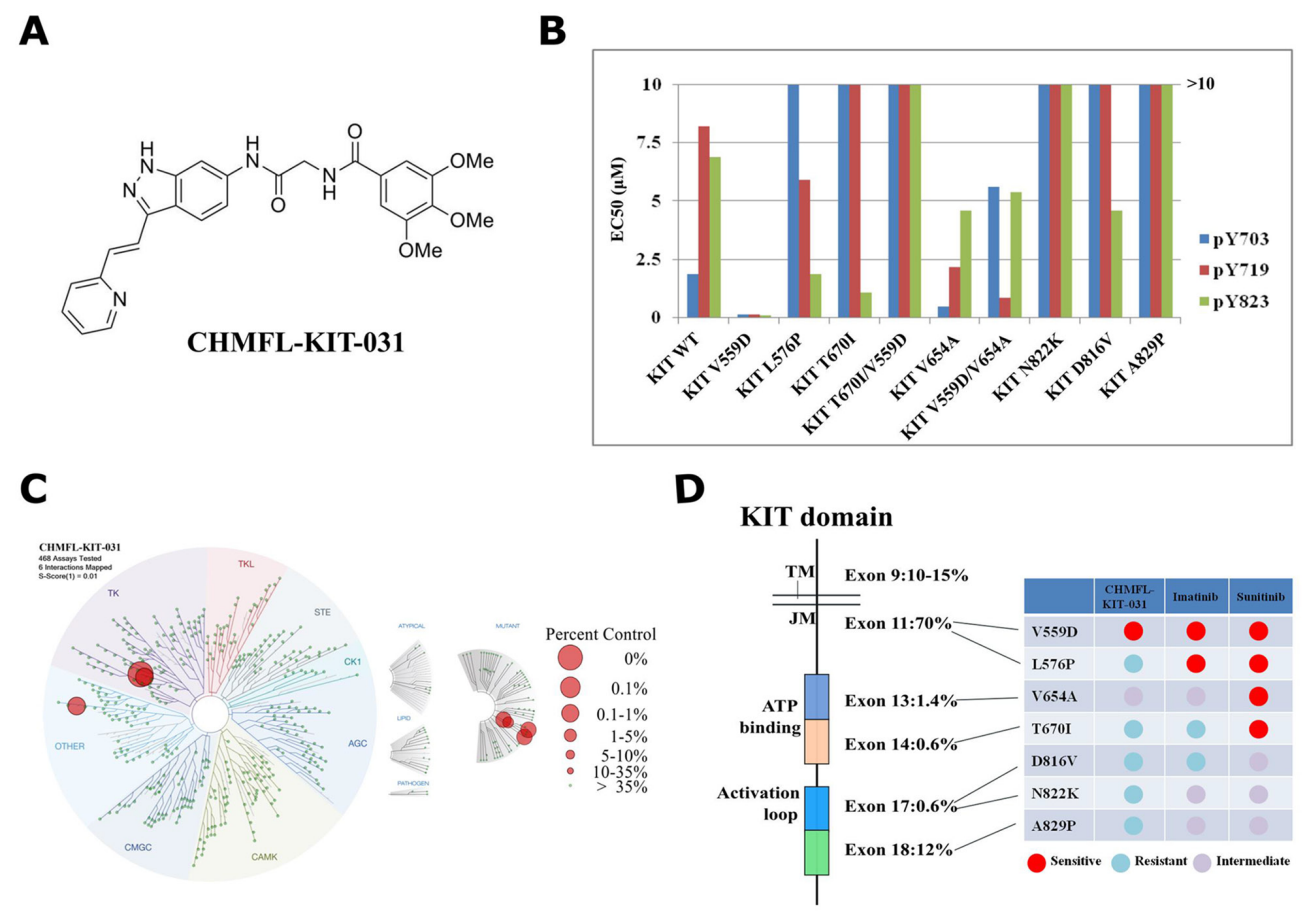
Characterization of CHMFL-KIT-031 as a KIT-V559D selective inhibitor **(A)** Chemical structure of CHMFL-KIT-031. **(B)** EC_50_ of CHMFL-KIT-031 for inhibition of auto-phosphorylation at KIT Y703, Y719 and Y823 sites on a panel of KIT mutants transformed BaF3 cells. **(C)** Kinomewide selectivity profiling of CHMFL-KIT-031 with KINOMEScan technology. **(D)** Schematic illustration of selectivity profile of CHMFL-KIT-031, Imatinib and Suninitib on the juxtamembrane domain primary mutants, ATP binding pocket secondary mutants and activation loop secondary mutants.

Through screening a panel of in-house made structure focused type II lead compounds prepared during development of KIT kinase inhibitors with KIT-V559D permanently transformed BaF3 cells [10], we found that CHMFL-KIT-031 (Figure 1A) was highly potent for inhibition of the proliferation of BaF3-TEL-KIT-V559D cells (GI_50_: 0.025 μM) (Table 1). Further testing demonstrated that it did not affect the parental BaF3 cells (GI_50_: >10 μM) which indicated that this compound displayed KIT V559D on-target anti-proliferative effect. Surprisingly, it did not affect the proliferation of KIT wt transformed BaF3 cells either (GI_50_: >10 μM) which indicated that it also achieved selectivity over KIT wt kinase. This encouraged us to expand the testing to other mutant isoforms. Interestingly, CHMFL-KIT-031 also did not affect L576P mutant occurred in the JM domain (GI_50_: 2.2 μM). It showed no apparent inhibitory activity against gatekeeper T670I mutant (ATP binding pocket), T670I/V559D (JM/ATP binding pocket) and activation loops mutants N822K, D816V (all GI_50_s: >10 μM). It only exhibited moderated inhibitory activity against ATP binding pocket mutant V654A (GI_50_: 0.4 μM) and V654A/V559D mixed mutant (GI_50_: 0.81 μM). Comparably, Imatinib was more sensitive against JM domain mutant V559D and L576P than KIT wt. In addition, Imatinib displayed similar sensitivities among KIT wt, V654A, V654A/V559D and N822K but resistant to KIT T670I, T670I/V559D and D816V, while Sunitinib exhibited similar potent activities against all of the tested mutants except that it was relatively resistant to KIT wt, N822K and D816V. Furthermore, Sunitinib also displayed inhibitory activity against parental BaF3 cells which again reflected its multi-target profiles.

**Table 1:** Antiproliferative effects of CHMFL-KIT-031 against a panel of KIT wt/mutants transformed BaF3 cells

GI_50_(μM)	Mutants locations	CHMFL-KIT-031	Imatinib	Sunitinib
BaF3	/	>10	>10	1.3
BaF3-tel-KIT	/	>10	0.59	0.06
BaF3-tel-KIT-V559D	JM domain	0.025	0.011	<0.0003
BaF3-tel-KIT-L576P	JM domain	2.2	0.007	0.003
BaF3-tel-KIT-T670I	ATP binding domain	>10	>10	0.001
BaF3-tel-KIT-T670I-V559D	JM/ATP binding domain	>10	5.2	0.007
BaF3-tel-KIT-V654A	ATP binding domain	0.4	0.51	0.002
BaF3-tel-KIT-V559D-V654A	JM/ATP binding domain	0.81	0.70	0.006
BaF3-tel-KIT-N822K	Activation loop	>10	0.41	0.12
BaF3-tel-KIT-D816V	Activation loop	>10	>10	0.42
BaF3-tel-KIT-A829P	Activation loop	1.8	0.48	0.13

### CHMFL-KIT-031 potently inhibits KIT auto-phosphorylation in BaF3-TEL-KIT-V559D cells

In order to confirm the selectivity observed in the anti-proliferation assay of the transformed BaF3 cells, we then examined the inhibitory effect of CHMFL-KIT-031 for the KIT wt/mutant auto-phosphorylation at Y703, Y719 and Y823 sites to further confirm the on-target efficacy (Figure 1B, Supplementary Figure 1 and Supplementary Table 1). The results showed that CHMFL-KIT-031 only potently affected KIT V559D mutant’s phosphorylation at Y703/719/823 sites but not others. This further confirmed that the anti-proliferative effect observed in the transformed BaF3 cells was achieved through isoform specific inhibition and hence the selectivity among different mutant isoforms was biologically relevant.

### CHMFL-KIT-031 exhibits high selectivity for KIT kinase in the kinome-wide selectivity profiling

Then we used the DiscoverX’s KINOMEScan platform [11] to further examine CHMFL-KIT-031’s kinome-wide selectivity profile. The results showed that it exhibited a great selectivity among 468 kinases/mutants at the concentration of 1 μM (S score (1)=0.01) (Figure 1C and Supplementary Table 2). Besides the strong binding to V559D, it also bound strongly to KIT wt, L576P and A829P. In addition, for other kinases it also displayed strong binding to CSF1R and NEK3 kinases. Given the fact that KINOMEScan is a competitive binding assay and sometimes it cannot really reflect the compound’s inhibitory activity, just like we have observed for KIT wt, L576P and A829P mutants which are not sensitive in the BaF3 cells (Table 1 and Figure 1B), we then tested it in the CSF1R transformed BaF3 cells and the results showed that there was no strong anti-proliferative effect (GI_50_: 2.4 μM). For NEK3 kinase there is no available biochemical assay yet, therefore we did not confirm it. Together, these results suggested that CHMFL-KIT-031 is a highly selective KIT V559D mutant inhibitor which exhibited a much better selectivity spectrum than Imatinib and Sunitinib (Figure 1D).

### CHMFL-KIT-031 selectively inhibits the KIT V559D mutant over KIT wt

**Figure 2:**
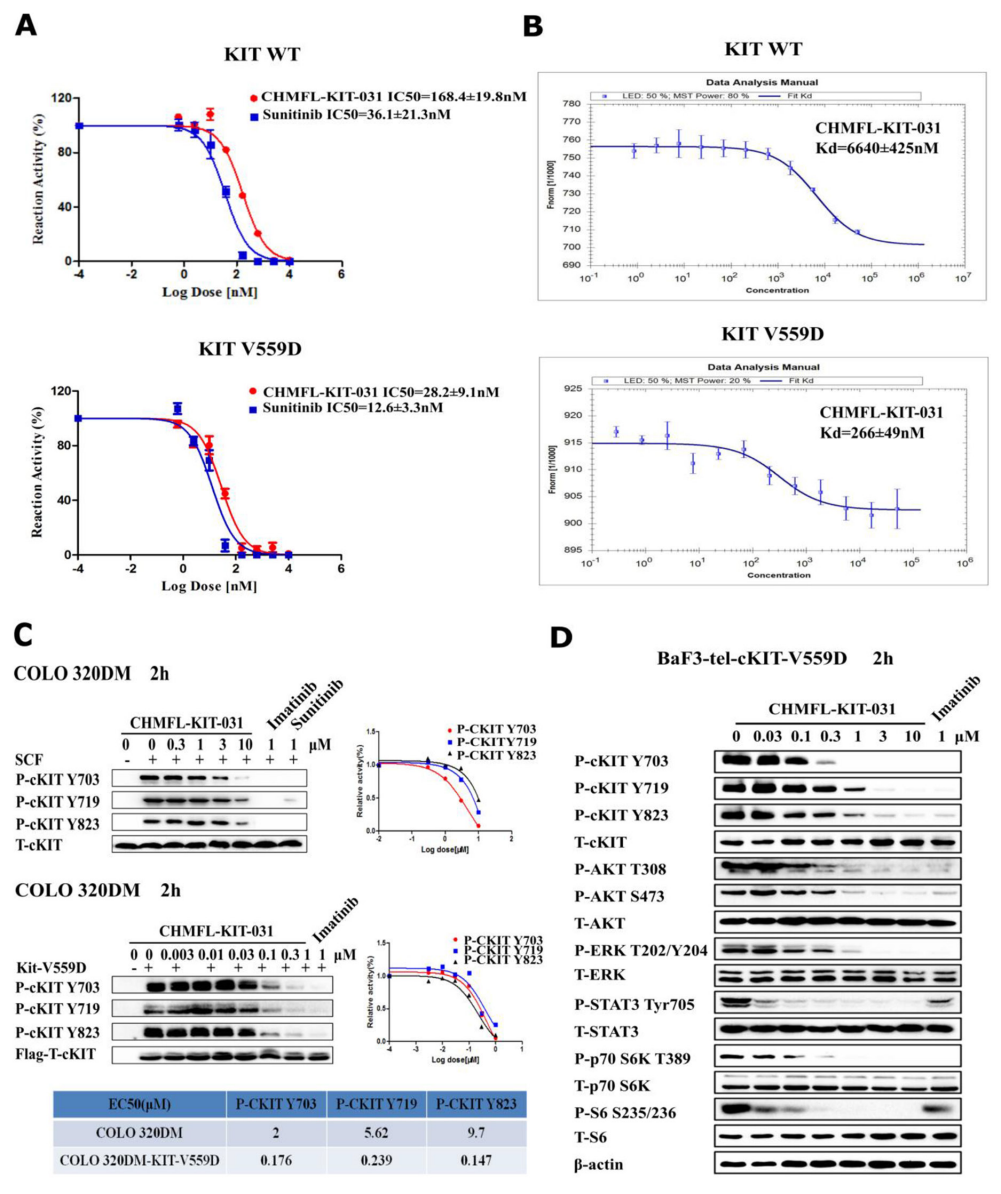
*In vitro* characterization of CHMFL-KIT-031 **(A)** IC_50_ determination of CHMFL-KIT-031 with purified KIT wt/V559D kinase protein using the ADP-Glo assay method. **(B)** Kd determination of CHMFL-KIT-031 with purified KIT wt/V559D kinase protein using the MST technology. **(C)** In cell EC_50_ determination of CHMFL-KIT-031 with parental Colo320DM (KIT wt) and KIT V559D overexpressed Colo320DM cells. **(D)** Effect of CHMFL-KIT-031 on KIT mediated signaling pathways in BaF3-TEL-KIT-V559D cells.

In order to better define CHMFL-KIT-031’s inhibitory effect against KIT V559D mutant, we then tested it with purified KIT wt/V559D kinase protein by ADP-Glo assay (Figure 2A). The results showed that it could potently inhibited KIT V559D (IC_50_: 28.2 nM) and displayed about 6-fold selectivity over KIT wt (IC_50_: 168.4 nM). In addition, we also examined its binding Kd using MST technology [12] and obtained Kd values of 266 nM to KIT V559D and 6640 nM to KIT wt (Figure 2B). We also transformed and overexpressed KIT V559D transiently into Colo320DM cells which express wt KIT kinase and tested CHMFL-KIT-031’s inhibitory effects for the auto-phosphorylation of KIT Y703, 719 and Y823 sites. Not surprisingly, it potently blocked the phosphorylation of KIT V559D mutant (EC_50_: ranging from 176 nM to 339 nM) and achieved over 10-fold selectivity compared to KIT wt at the corresponding phosphorylation sites (Figure 2C). In addition, in the KIT-V559D transformed stable BaF3 cells, the inhibitor not only affected the phosphorylation of KIT, but also potently inhibited the downstream mediators such as AKT, S6, ERK, p70S6K and STAT3’s phosphorylation (Figure 2D). These results further suggested that the selectivity of CHMFL-KIT-031 is biologically relevant.

### CHMFL-KIT-031 inhibits the tumor growth in BaF3-TEL-KIT-V559D cell inoculated *in vivo* model

**Figure 3:**
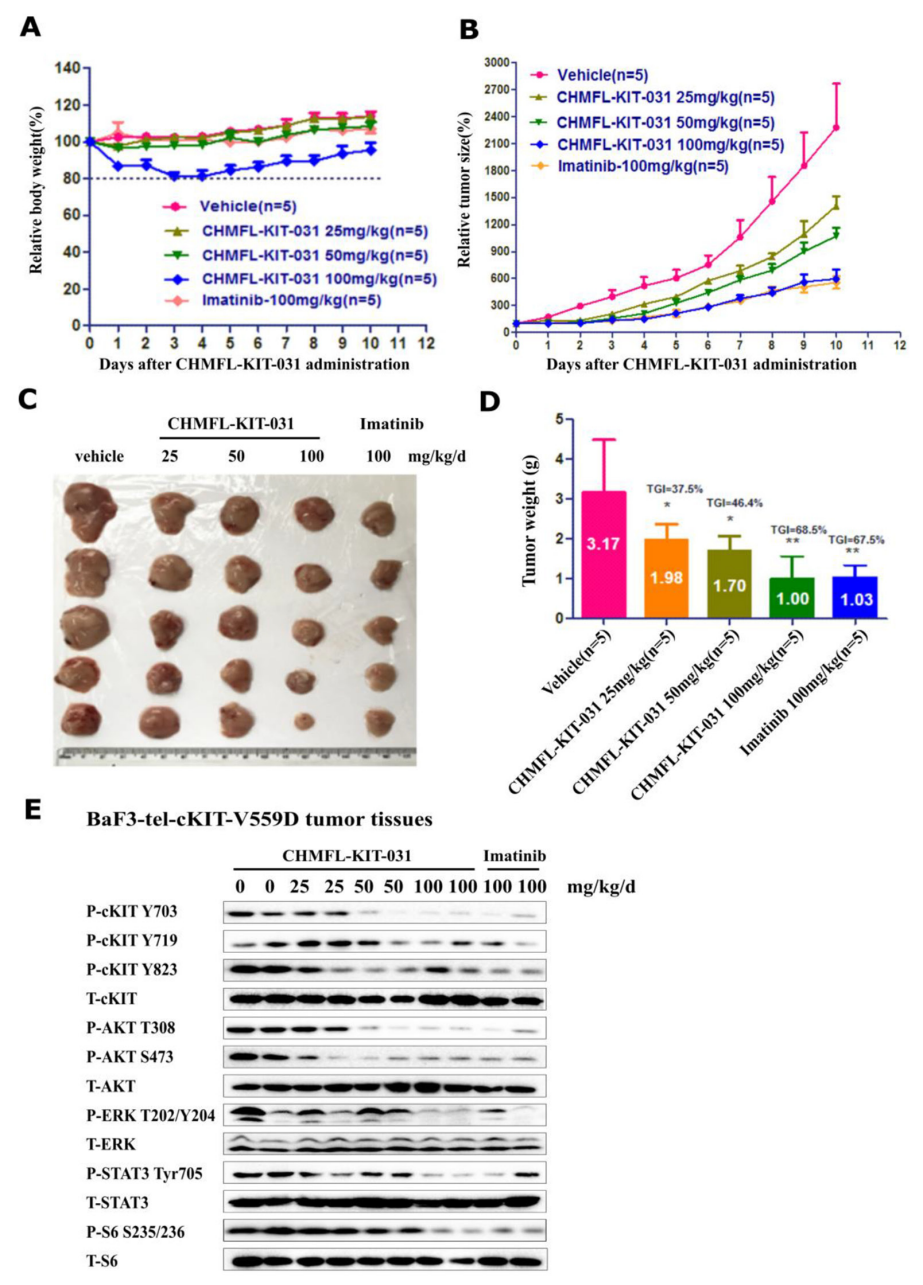
Effect of CHMFL-KIT-031 in the BaF3-TEL-KIT-V559D inoculated mouse model Female nu/nu mice bearing established BaF3-TEL-KIT-V559D tumor allagraft model were treated with CHMFL-KIT-031 at 25, 50 and 100 mg/kg/d dosages or vehicle. Daily IP administration was initiated when BaF3-TEL-KIT-V559D tumors had reached a size of 200 to 400 mm^3^. Each group contained five animals. Data, mean ± SEM. **(A)** Body weight and **(B)** Tumor size measurements from BaF3-TEL-KIT-V559D allagraft mice after 10 days CHMFL-KIT-031 administration. Initial body weight and tumor size were set as 100%. **(C)** Representative photographs of tumors in each group after 0, 25, 50 and 100 mg/kg/d CHMFL-KIT-031 or vehicle treatment. **(D)** Comparison of the final tumor weights in each group after 10-day treatment period of CHMFL-KIT-031. Numbers in columns indicate the mean tumor weight in each group. ns, p>0.05, (^*^) p<0.05, (^**^) p<0.01. **(E)** Western blot analysis of CHMFL-KIT-031 on KIT signaling pathways in the tumor tissues after 10-day treatment period.

We then tested CHMFL-KIT-031 in the BaF3-TEL-KIT-V559D inoculated mouse model. Through IP injection, the compound displayed a dose-dependent slow down of the tumor progression and 100 mg/kg/day treatment achieved 68.5% tumor growth inhibition (TGI) which was similar to the multi-target inhibitor Imatinib (Figure 3A-3D). Western blot analysis of the cells extracted from the tumor tissue showed that starting from 50 mg/kg dosage it could significantly affect KIT, AKT, S6 and STAT3’s phosphorylation and the effect was more clear at 100 mg/kg dosage, which is the same with Imatinib (Figure 3E). Immunohistochemistry (IHC) staining of the tumor tissue also confirmed that the cell proliferation was inhibited and the apoptosis was induced in the tumor tissue (Supplementary Figure 2).

## DISCUSSION

Primary gain-of-function mutations of kinase are usually the driving oncogenic force for tumorigenesis. Most of the time this kind of mutations would either induce the conformational change or the Km change of ATP, which will lead to constitutive kinase activation. It is still unclear how such mutations cause this consequence although this is a basic biochemistry question. One of the hurdles for understanding this question is that it is hard to determine how such mutations induce the conformational change due to the unstable superactive state of the protein. For example, we have tried different methods to obtain the X-ray structure of the KIT V559D mutant but failed due to the instability. In addition, such mutations usually do not occur in the well-defined ATP binding pocket, which makes the theoretical calculation and prediction very hard. And this would also make it difficult for the rational design of the mutant isoform selective inhibitors. However, a mutant selective inhibitor would undoubtedly help to better understand the mechanism of the biological consequences stimulated by these primary gain-of-function mutations.

From the potential clinical application point of view, a highly selective KIT V559D inhibitor would be highly desired. Compared with the current clinically used front line GIST’s target therapy imatinib, CHMFL-KIT-031 completely abolished the inhibitory activity against ABL kinase, which has normal physiological functions in other organs. This could alleviate the potential off-target mediated side effects and hence increase the therapeutic window. In addition, in GISTs it is the mutant KIT kinase isoform such as V559D that makes the gene oncogenic but not the wide type, meanwhile the normal KIT wide type plays critical roles in hematopoietic stem cells. Therefore CHMFL-KIT-031 which achieved selectivity between prevalent primary gain-of-function KIT mutant over the KIT wide type would provide further mechanism based safety advantages.

In summary, through a high throughput screening approach, we have discovered a highly KIT V559D mutant selective and potent kinase inhibitor CHMFL-KIT-031, which exhibited 10-fold selectivity over KIT wt and did not potently inhibit other KIT primary and secondary mutants. In addition, it also achieved high selectivity over other kinases in the kinome. The *in vitro* and *in vivo* characterizations have proved that the inhibitor’s selectivity and potency are biologically relevant. Given the fact that KIT V559D is the most prevalent primary gain-of-function mutation in the GISTs, CHMFL-KIT-031 would be a valuable pharmacological tool to help to dissect its pathological roles. In addition, from the drug discovery point of view, more preclinical evaluation would be needed for tailoring its drug-like properties which is required for moving this type of inhibitors into the clinical investigation.

## MATERIALS AND METHODS

### Reagents and antibodies

CHMFL-KIT-031(chemical structure shown in Figure 1A) were synthesized in our laboratory (details in Supplementary material). Imatinib and Sunitinib were purchased from Haoyuan Chemexpress Inc (shanghai, P.R.china). Antibodies used for rabbit polyclonal antibodies to total c-KIT (cat. no. 3308) and phospho-KIT Y719 (cat. no. 3391) and Y703 (cat. no. 3073) were purchased from Cell Signaling Technology. Rabbit polyclonal antibodies to phospho-KIT Y823 was from Invitrogen (cat. no. 44−498G). Polyclonal rabbit antibodies total p42/44 mitogen activated protein kinase (MAPK) (cat. no. 4695), phospho-p44/42 MAPK T202/Y204 (cat. no. 4370), phospho-AKTS473 (cat. no. 4060), total AKT (cat. no. 4691), phospho-Stat3 (cat. no. 9145), totalStat3 (cat. no. 12640), phospho-S6K T389 (cat. no. 9206), total S6K (cat. no. 9202), phospho-S6 S235/236 (cat. no. 2211), total S6 (cat. no. 2217) were from Cell Signaling Technology. Beta actin antibodies were purchased from Sigma (cat. no. A5316).

### Cell culture and KIT wt/mutants transformed stable BaF3 cells generation

The KIT mutant isogenic BaF3 cells lines and Colo320DM cells were cultured in RPMI 1640 media (Corning, USA) with 10% fetal bovine serum (FBS) and supplemented with 2% L-glutamine 1% penicillin/streptomycin, the BaF3 cell line was cultured in RPMI 1640 media (Corning, USA) with 1% IL-3, 10% fetal bovine serum (FBS) and supplemented with 2% L-glutamine 1% penicillin/streptomycin. All cell lines were maintained in culture media at 37°C with 5% CO_2_.

Retroviral constructs for BaF3-KIT mutants were made based on the pMSCVpuro (Clontech) backbone. For TEL-KIT vector, the first 1 kb of human TEL gene with an artificial myristoylation sequence (MGCGCSSHPEDD) was cloned into the pMSCVpuro retroviral vector, followed by a 3xFLAG tag sequence. Then, the kinase domain coding sequence of KIT was inserted in-frame between TEL and 3xFLAG sequences. For full-length expression vectors, the coding sequences of KIT variants were directly cloned in pMSCVpuro vector with a 3xFLAG tag at the C-terminal end. All mutagenesis was performed using the QuikChange Site-Directed Mutagenesis Kit (Stratagene) following the manufacturer’s instructions. Retrovirus was packaged in HEK293T cells by transfecting KIT-containing MSCV vectors together with two helper plasmids. Virus supernatants were harvested 48 h after transfection and filtered before infection. Then BaF3 cells were infected with harvested virus supernatants using spinoculation protocol, and stable cell lines were obtained by puromycin selection for 48 h. The IL-3 concentrations in the culture medium were gradually withdrawn until cells were able to grow in the absence of IL-3.

### Anti-proliferation assay of KIT wt/mutants transformed stable BaF3 cells

Cells were grown in 96-well culture plates (2500/well). The compounds of various concentrations were added into the plates. Cell proliferation was determined after treatment with compounds for 72 hours. Cell viability was measured using the CellTiter–Glo assay (Promega, USA), according to the manufacturer’s instructions, and luminescence was measured in a multi-label reader (Envision, PerkinElmer, USA). Data were normalized to control groups (DMSO) and represented by the mean of three independent measurements with standard error <20%. GI_50_ values were calculated using Prism 5.0 (GraphPad Software, San Diego, CA).

### KIT wt/V559D protein expression

The sequences encoding wt KIT residues 544-935 with a His tag were cloned into baculovirus expression vector pFASTHTA. The proteins were expressed by infecting SF9 cells with high titer viral stocks for 48h. Cells were harvested and lysed in 25mM Tris pH7.4, 250mM NaCl, 1mM PMSF. The supernatant was loaded to Ni-NTA Column (QIAGEN, 1018244). Then the proteins were step eluted with the same buffer with 250mM imidazole. The eluted proteins were loaded on a Superdex-200 column equilibrated in 25mM Tris (pH 7.4), 250mM NaCl, 1mM DTT, and 1mM EDTA. Peak fractions were concentrated to 2mg/mL, and flash-frozen.

Kit V559D encoding residues 545-976 with His tag was cloned into baculovirus expression vector pFAST-HTA. The recombinant bacmid was transfected into SF9 by cellfectin (Invitrogen). High titer viral stocks were obtained by two rounds of amplification of the virus. The protein was expressed by infecting SF9 cells with high titer viral stocks for 48 h. Cells were harvested and resuspended in lysis buffer (25 mM Tris pH 8.0, 250mM NaCl, and 1 mM PMSF). The cells were lysed by ultrasonication, and the cell debris was removed by ultracentrifugation. The supernatant was incubated with Ni-affinity beads (GE). The beads were then washed by lysis buffer containing 50−250 mM imidazole. The elute was loaded to Sephedex 75. The protein was concentrated to 0.6 mg/mL, and aliquots were frozen and stored at −80°C.

### ADP-Glo biochemical assay

The ADP-Glo kinase assay (Promega, Madison, WI, USA) was used to screen CHMFL-KIT-031 for its c-KIT and the relevant mutations inhibition effects. The kinase reaction system contains 9 μL c-KIT (20 ng/μL) or c-KIT V559D (15 ng/μL), 1 μL of serially diluted CHMFL-KIT-031, and 10 μL substrate Poly (4:1 Glu, Tyr) peptide (0.4 μg/μL) (Promega, Madison, WI) with 100 μM ATP (Promega, Madison, WI). The reaction in each tube was started immediately by adding ATP and kept going for an hour under 37°C. After the tube cooled for 5 minutes at room temperature, 5 μL solvent reactions were carried out in a 384-well plate. Then 5 μL of ADP-Glo reagent was added into each well to stop the reaction and consume the remaining ATP within 40 minutes. At the end, 10 μL of kinase detection reagent was added into the well and incubated for 30 minutes to produce a luminescence signal. Luminescence signal was measured with an automated plate reader (Envision, PE, USA) and the dose-response curve was fitted using Prism 5.0 (GraphPad Software Inc., San Diego, CA, USA). The biochemical tests of other targets were provided by Invitrogen (Carlsbad, CA, USA).

### Kd determination by MST

The Kd values were measured using the Monolith NT.115 instrument (NanoTemper Technologies). The proteins were fluorescently labeled according to the manufacturer’s protocol. A range of concentrations of compound CHMFL-KIT-031 (from 0.05 mM to 2.5 nM) were incubated with 200 nM of purified labeled KIT-V559D and wt-KIT protein for 10 min in assay buffer (50mM Tris, 150mM NaCl, pH7.5, and 0.05% Tween 20). The sample was loaded into the NanoTemper glass capillaries and microthermophoresis was carried out using 50% LED power,20% MST power for KIT-V559D and 50% LED power, 80% MST power for wt-KIT. The Kd values were calculated using the mass action equation via the NanoTemper software from duplicate reads of triplicate experiments.

### Colo320DM-KIT V559D transient overexpression cells

KIT-V559D kinase domain coding sequence was inserted into a pMSCV puro vector (Clontech) that was modified by inserting a 3×FLAG sequence at the 3’ end of the multicloning sites. The vector was transiently transfected into Colo320DM cells with TransIN^™^EL transfection reagent. Colo320DM-KIT V559D transient overexpression cells were harvested 48 h after transfection.

### Signaling pathway studies

The KIT mutant isogenic BaF3 cells and Colo320DM-KIT V559D transient overexpression cells were treated with DMSO, serially diluted CHMFL-KIT-031 and 1μM Imatinib for 2h. Cells were washed in 1×PBS buffer and lysed in cell lysis buffer at 4°C for 30 min. The lysates were cleared by centrifugation, and the protein concentrations were measured by BCA analysis (Beyotime, China). Lysates containing 50 μg of total proteins were fractionated on a 10% SDS polyacrylamide gel (Bio-Rad) and transferred to nitrocellulose Membrane (sartorius stedim). Incubation of primary antibodies was performed overnight at 4°C. Colo320DM cells were incubated DMSO, serially diluted CHMFL-KIT-031, 1μM Imatinib and1μM Sunitinib for 2h before 10 ng/mL SCF (R&D Systems) stimulation. After SCF stimulation, we utilized the same method as previously described.

### BaF3-TEL-KIT-V559D cell mediated animal study

#### BaF3-TEL-KIT-V559D allograft model

Five-week-old female nu/nu mice were purchased from Nanjing Biomedical Research Institute of Nanjing University (Nanjing, China). All animals were housed in a specific pathogen-free facility and used according to the animal care regulations of Hefei Institutes of Physical Science, Chinese Academy of Sciences (Hefei, China). Prior to implantation, cells were harvested during exponential growth. One million BaF3-TEL-KIT-V559D cells in PBS were formulated as a 1:1 mixture with Matrigel (BD Biosciences) and injected into the subcutaneous space on the right flank of nu/nu mice. Daily ip injection was initiated when BaF3-TEL-KIT-V559D tumors had reached a size of 200−400 mm^3^. Animals were then randomized into treatment groups of 5 mice each for efficacy studies. CHMFL-KIT-031 was delivered daily in a HKI solution (0.5% methocellulose/0.4% Tween80 in ddH_2_O) by ip injection. A range of doses of CHMFL-KIT-031 or its vehicle were administered, as indicated in the Figure 3 caption. Body weight and tumor growth was measured daily after CHMFL-KIT-031 treatment. Tumor volume was calculated as follows: tumor volume (mm^3^) = [(W^2^ × L)/2] in which width (W) is defined as the smaller of the two measurements and length (L) is defined as the larger of the two measurements.

#### HE staining

HE staining was carried out according to previous publication. First, the sections were hydrated and then the slide was dipped into a Coplin jar containing Mayer’s hematoxylin and agitated for 30 s. After rinsing the slide in H_2_O for 1 min, it was stained with 1% eosin Y solution for 10−30 s with agitation. Subsequently, the sections were dehydrated with two changes of 95% alcohol and two changes of 100% alcohol for 30 s each, and then the alcohol was extracted with two changes of xylene. Finally, one or two drops of mounting medium was added and covered with a coverslip.

#### *K*_i_-67 staining

For IHC demonstration of K_i_-67, tissue sections were quenched for endogenous peroxides and placed in an antigen retrieval solution (0.01 M citrate buffer, PH 6.0) for 15 min in a microwave oven at 100°C at 600 W. After incubation in the casein block, mouse mAb anti-K_i_-67 (ZSGB-BIO, China) was applied to the sections at dilutions of 1:50. Incubations with primary antibodies lasted overnight at 4°C. The secondary detection system was used to visualize antibody binding. Staining was developed with DAB, and the slides were counterstained with hematoxylin, dehydrated and mounted.

#### TUNEL staining

TUNEL staining was performed using the POD in situ cell death detection kit (Roche, USA). Briefly, sections were deparaffinized in xylene, rehydrated in decreasing concentration of ethanol, and then treated by nuclease free proteinase K for 15 min at room temperature before endogenous peroxidase was blocked in 3% H_2_O_2_ in methanol. Terminal deoxynucleotidyl transferase (TdT) in reaction buffer was applied to sections for 1 h at 37°C. Following washes, the slides were covered by converter-POD solution for 30 min at 37°C. Apoptotic cells were detected after incubation in 3,3’-diaminobenzidine (DAB) chromogen (Beyotime Biotechnology, China) for approximately 8 min, and the slides were counterstained with hematoxylin.

### Appendix A: supplementary data

Supplementary methods and data can be found with this article online available.

## SUPPLEMENTARY MATERIALS FIGURES AND TABLES




