# Supplementary material for: Discovery of a highly selective KIT kinase primary V559D mutant inhibitor for gastrointestinal stromal tumors (GISTs)
Source: Oncotarget. 2017 Nov 15;8(67):111110–8. doi: 10.18632/oncotarget.22624 (PMC5762309; doi:10.18632/oncotarget.22624)
Supplement: Supplementary file 1 [file oncotarget-08-111110-s001.pdf]

Discovery of a highly selective KIT kinase primary V559D mutant inhibitor for gastrointestinal stromal tumors (GISTs)

SUPPLEMENTARY MATERIALS

General information

All reagents and solvents were purchased from commercial sources and used as obtained. ^1H NMR and ^{13}C NMR spectra were recorded with a Bruker 400 NMR spectrometer and referenced to deuterium dimethyl sulfoxide (DMSO-d_6). Chemical shifts are expressed in ppm. In the NMR tabulation, s indicates singlet; d, doublet; t, triplet; q, quartet; m, multiplet; and br, broad peak. LC/MS were performed on *an* Agilent 6224 TOF using an ESI source coupled to an Agilent 1260 Infinity HPLC system operating in reverse mode with an Agilent XDB-C18 column (4.6 × 50 mm, 1.8 µm) using a water/acetonitrile (each with 0.2% (v/v) formic acid) gradient at a flow rate at 0.4 mL/min. Flash column chromatography was conducted using silica gel (Silicycle 40−64 µm). The purities of all compounds were determined to be above 95% by HPLC.

Chemical synthesis and characterization of CHMFL-KIT-031

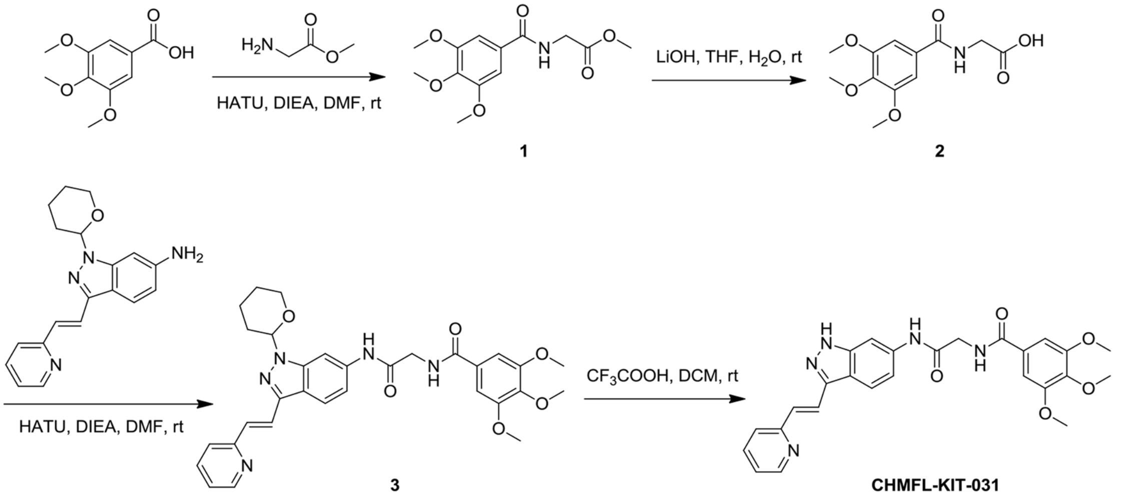

Methyl (3,4,5-trimethoxybenzoyl)glycinate (1) To a solution of 3,4,5-trimethoxybenzoic acid (1.1 g, 5.2 mmol) in DMF (20 mL) were added glycine methyl ester hydrochloride (0.8 g, 6.4 mmol), HATU (3.0 g, 7.8 mmol), and DIEA (1.3 g, 10.4 mmol). The resulting mixture was stirred at room temperature for 30 min. Then it was diluted with EtOAc (150 mL), washed with water (100 mL× 3) and brine (100 mL). The organic layers were dried over anhydrous sodium sulfate, concentrated and purified by silica gel flash chromatography (0−2% MeOH in DCM) to afford compound **1** (1.2 g, 82%) as a white solid.

^1H NMR (400 MHz, DMSO-d_6): δ 8.94 (s, 1H), 7.23 (s, 2H), 4.03 (s, 2H), 3.83 (s, 6H), 3.72 (s, 3H), 3.66 (s, 3H). ^{13}C NMR (100 MHz, DMSO-d_6): δ 170.92, 166.48, 153.09, 140.67, 129.17, 105.32, 60.53, 56.43, 52.17, 41.75. LC-MS (ESI, m/z): calcd for $C_{13}H_{18}NO_6$ $[M + H]^+$, 284.1129; found 284.1133.

(3,4,5-Trimethoxybenzoyl)glycine (2) To a solution of **1** (1.0 g, 3.5 mmol) in THF (15 mL) were added LiOH (0.8 g, 35.3 mmol) and water (15 mL). The reaction mixture was stirred at room temperature for 2 h under argon protection. The mixture was acidified with hydrochloric acid and the solution was extracted with ethyl acetate (100 mL). The organic solution was washed with diluted hydrochloric acid (50 mL) and brine (50 mL). The organic layers were dried over anhydrous sodium sulfate, and evaporated to give the title compound **2** (0.8 g, 85%) as a white solid.

^1H NMR (400 MHz, DMSO-d_6): δ 8.85 (s, 1H), 7.23 (s, 2H), 3.93 (s, 2H), 3.83 (s, 6H), 3.71 (s, 3H). ^{13}C NMR (100 MHz, DMSO-d_6): δ 171.88, 166.32, 153.05, 140.55, 129.49, 105.31, 60.53, 56.44, 41.80. LC-MS (ESI, m/z): calcd for $C_{12}H_{16}NO_6$ $[M + H]^+$, 270.0972; found 270.0973.

(*E*)-3,4,5-Trimethoxy-*N*-(2-oxo-2-((3-(2-(pyridin-2-yl)vinyl)-1-(tetrahydro-2*H*-pyran-2-yl)-1H-indazol-6-yl)amino)ethyl)benzamide (3) To a solution of 2 (0.6 g, 2.2 mmol) in DMF (15 mL) were added (*E*)-3-(2-(pyridin-2-yl)vinyl)-1-(tetrahydro-2H-pyran-2-yl)-1H-indazol-6-amine (0.9 g, 2.7 mmol), HATU (1.3 g, 3.3 mmol) and

DIEA (0.6 g, 4.4 mmol). The resulting mixture was stirred at room temperature for 30 min. Then it was diluted with ethyl acetate (120 mL), washed with water (80 mL× 3) and brine (80 mL). The organic layers were dried over anhydrous sodium sulfate, concentrated and purified by silica gel flash chromatography (0−5% MeOH in DCM) to afford compound 3 (1.1 g, 88%) as a white solid.

^{1}H NMR (400 MHz, DMSO-d_6): δ 10.39 (s, 1H), 8.90 (s, 1H), 8.62 (s, 1H), 8.27 (s, 1H), 8.16 (d, J = 8.0 Hz, 1H), 7.90 (d, J = 16.4 Hz, 1H), 7.81 (s, 1H), 7.69 (s, 1H), 7.59 (d, J = 16.4 Hz, 1H), 7.39 (s, 1H), 7.29 (m, 3H), 5.75 (s, 1H), 4.16 (s, 2H), 3.86 (m, 7H), 3.73 (m, 4H), 2.45 (s, 1H), 2.03 (s, 2H), 1.77 (s, 1H), 1.59 (s, 2H). ^{13}C NMR (100 MHz, DMSO-d_6): δ 168.77, 166.60, 155.26, 153.07, 150.04, 141.75, 141.51, 140.58, 138.41, 137.34, 130.32, 129.59, 123.84, 123.14, 123.04, 121.72, 118.87, 115.94, 105.43, 100.10, 84.96, 67.10, 60.57, 56.47, 43.96, 29.27, 25.29, 22.50. LC-MS (ESI, m/z): calcd for $C_{31}H_{34}N_5O_6$ [M + H]$^+$, 572.2504; found 572.2506.

CHMFL-KIT-031. Compound **3** (1.0 g, 1.8 mmol) was dissolved in dichloromethane (8 mL) and TFA (8 mL) and the mixture was stirred at room temperature for 1 hour. The reaction mixture was then concentrated in vacuo and acidified with saturated aqueous sodium bicarbonate and the solids were filtered, washed with water (200 mL) and dried at 40° C for 48 h to provide **CHMFL-KIT-031** (0.8 g, 91%) as a yellow solid.

^{1}H NMR (400 MHz, DMSO-d_6): δ 13.20 (s, 1H), 10.33 (s, 1H), 8.90 (s, 1H), 8.63 (s, 1H), 8.19 (s, 1H), 8.14 (s, 1H), 7.98 (s, 1H), 7.92 (d, J = 16.6 Hz, 1H), 7.77 (s, 1H), 7.56 (d, J = 16.6 Hz, 1H), 7.31 (d, J = 27.6 Hz, 4H), 4.13 (s, 2H), 3.85 (s, 6H), 3.73 (s, 3H). ^{13}C NMR (100 MHz, DMSO-d_6): δ 168.75, 166.60, 155.00, 153.07, 149.13, 142.48, 142.02, 140.57, 138.21, 138.02, 129.59, 128.21, 125.51, 123.09, 123.03, 121.38, 117.55, 115.34, 105.43, 99.79, 60.56, 56.46, 43.99. LC-MS (ESI, m/z): calcd for $C_{26}H_{26}N_5O_5$ [M + H]$^+$, 488.1928; found 488.1930.

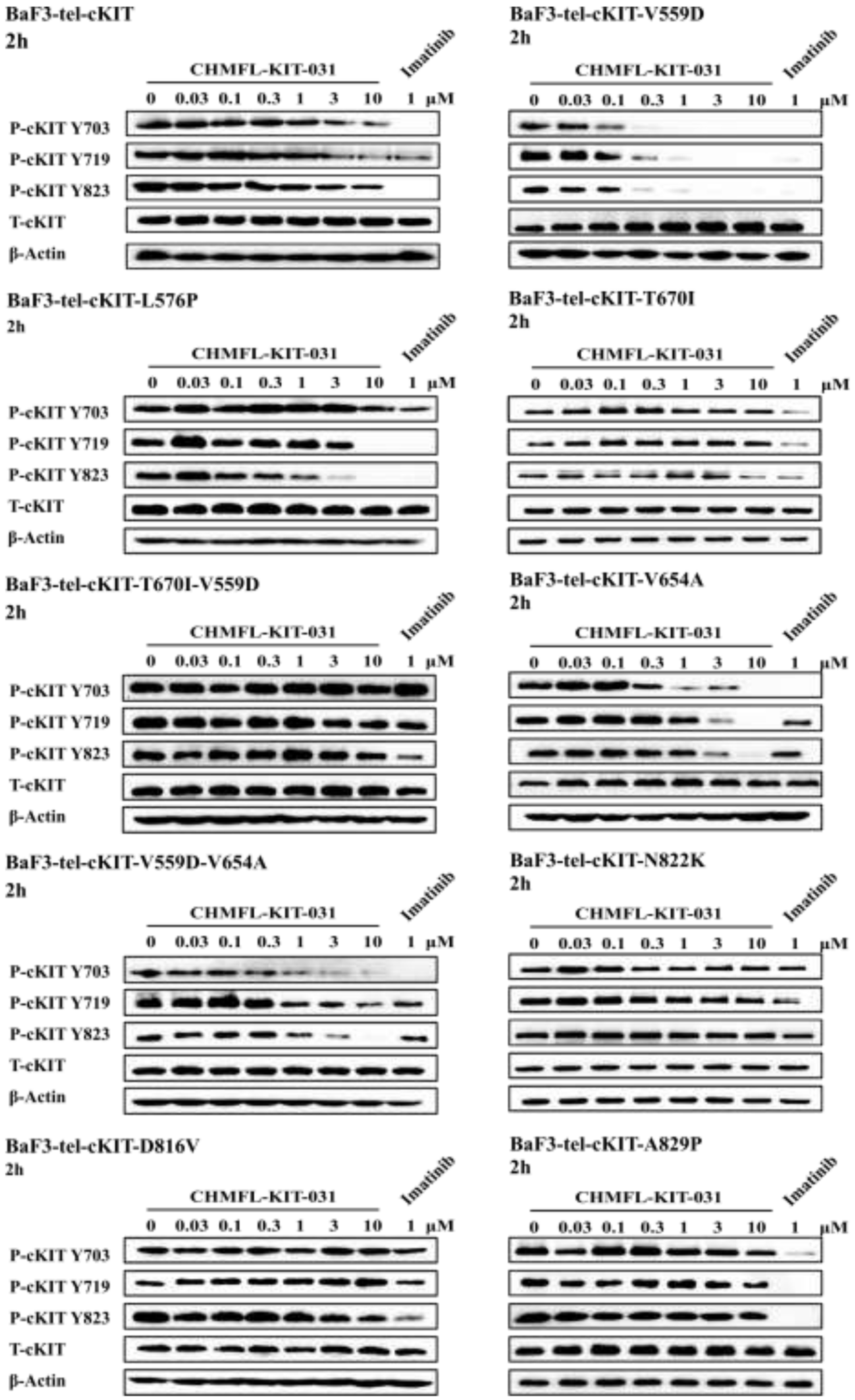

Supplementary Figure 1: EC_{50} determination of CHMFL-KIT-031 in KIT wt/mutants transformed BaF3 cells.

Supplementary Table 1: Inhibition Effects ofCHMFL-KIT-031 on the auto phosphorylationof Y703, Y719 and Y823 in KIT wt/mutants transformed BaF3 cells

KIT mutants (EC_{50}: µM)	pY703	pY719	pY823
KIT wt	1.9	8.2	6.9
KIT V559D	0.151	0.137	0.118
KIT L576p	>10	5.9	1.9
KIT T670I	>10	>10	1.1
KIT T670I/V559D	>10	>10	>10
KIT V654A	0.508	2.2	4.6
KIT V559D/V654A	5.6	0.863	5.4
KIT N822K	>10	>10	>10
KIT D816V	>10	>10	4.59
KIT A829P	>10	>10	>10

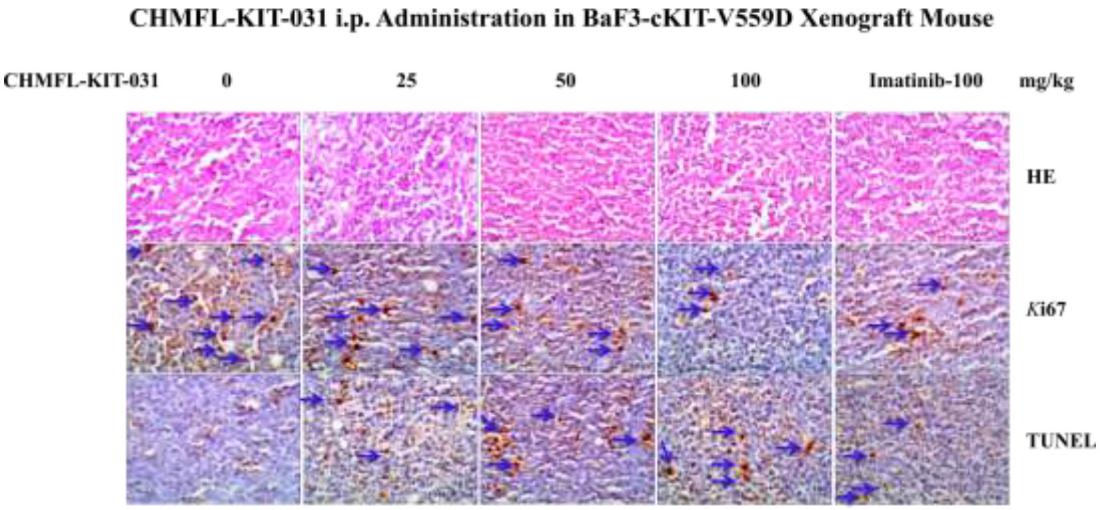

Supplementary Figure 2: IHC staining of the tumor tissues after CHMFL-KIT-031 treatment.

Supplementary Table 2: Kinome wide selectivity profiling of CHMFL-KIT-031 with DiscoverX's KINOMEScan™ technology

See Supplementary File 1
